# Immunodominant fragments of myelin basic protein initiate T cell-dependent pain

**DOI:** 10.1186/1742-2094-9-119

**Published:** 2012-06-07

**Authors:** Huaqing Liu, Sergey A Shiryaev, Andrei V Chernov, Youngsoon Kim, Igor Shubayev, Albert G Remacle, Svetlana Baranovskaya, Vladislav S Golubkov, Alex Y Strongin, Veronica I Shubayev

**Affiliations:** 1 Department of Anesthesiology, University of California, San Diego, 9500 Gilman Dr., Mail Box 0629, La Jolla, CA, 92093-0629, USA; 2 VA San Diego Healthcare System, La Jolla, CA, USA; 3 Sanford-Burnham Medical Research Institute, La Jolla, CA, USA; 4 Agilent Technologies, La Jolla, CA, USA

## Abstract

**Background:**

The myelin sheath provides electrical insulation of mechanosensory Aβ-afferent fibers. Myelin-degrading matrix metalloproteinases (MMPs) damage the myelin sheath. The resulting electrical instability of Aβ-fibers is believed to activate the nociceptive circuitry in Aβ-fibers and initiate pain from innocuous tactile stimulation (mechanical allodynia). The precise molecular mechanisms, responsible for the development of this neuropathic pain state after nerve injury (for example, chronic constriction injury, CCI), are not well understood.

**Methods and results:**

Using mass spectrometry of the whole sciatic nerve proteome followed by bioinformatics analyses, we determined that the pathways, which are classified as the Infectious Disease and T-helper cell signaling, are readily activated in the nerves post-CCI. Inhibition of MMP-9/MMP-2 suppressed CCI-induced mechanical allodynia and concomitant TNF-α and IL-17A expression in nerves. MMP-9 proteolysis of myelin basic protein (MBP) generated the MBP84-104 and MBP68-86 digest peptides, which are prominent immunogenic epitopes. In agreement, the endogenous MBP69-86 epitope co-localized with MHCII and MMP-9 in Schwann cells and along the nodes of Ranvier. Administration of either the MBP84-104 or MBP68-86 peptides into the naïve nerve rapidly produced robust mechanical allodynia with a concomitant increase in T cells and MHCII-reactive cell populations at the injection site. As shown by the genome-wide expression profiling, a single intraneural MBP84-104 injection stimulated the inflammatory, immune cell trafficking, and antigen presentation pathways in the injected naïve nerves and the associated spinal cords. Both MBP84-104-induced mechanical allodynia and characteristic pathway activation were remarkably less prominent in the T cell-deficient athymic nude rats.

**Conclusions:**

These data implicate MBP as a novel mediator of pain. Furthermore, the action of MMPs expressed within 1 day post-injury is critical to the generation of tactile allodynia, neuroinflammation, and the immunodominant MBP digest peptides in nerve. These MBP peptides initiate mechanical allodynia in both a T cell-dependent and -independent manner. In the course of Wallerian degeneration, the repeated exposure of the cryptic MBP epitopes, which are normally sheltered from immunosurveillance, may induce the MBP-specific T cell clones and a self-sustaining immune reaction, which may together contribute to the transition of acute pain into a chronic neuropathic pain state.

## Background

Pain is typically mediated by small unmyelinated C-nociceptive and thinly myelinated Aδ-afferents. Non-nociceptive, large-diameter myelinated Aβ-afferents transmit touch and vibration sense. However, following damage to the peripheral nervous system (PNS), Aβ-afferents join nociceptive circuitry [1]. Our mechanistic understanding of why the damaged Aβ-afferents interpret an innocuous, low-threshold tactile stimulus as painful (that is, mechanical allodynia) remains exceedingly limited. Growing evidence supports a model in which the damage to the electrically insulating myelin sheath and the resulting loss of electrical stability in Aβ-afferents contribute to the development of mechanical allodynia [2-6].

Mechanical allodynia and other forms of neuropathic pain (NP, that is, pain arising as a direct consequence of a lesion or disease affecting the somatosensory nervous system [7]) have features of a neuroimmune disorder [8]. T lymphocyte infiltration into both the damaged nerve [9-11] and the spinal cord at a corresponding segment [12-14] has been implicated in NP. Following chronic constriction injury (CCI), T cell-deficient athymic nude rats exhibit a diminished ability to develop NP, which is reversed with adoptive transfer of T-helper (Th)1 cells [9]. Interleukin (IL)-17, expressed by certain Th cells, is essential to mediating mechanical, but not thermal, hypersensitivity [15]. Major histocompatibility complex (MHC) II, produced by antigen-presenting cells to capture and present antigens to T cells, is required for the development of NP [16,17]. An increase in MHCII is observed in the nerve and the corresponding DRG [18], spinal cord [14,16], and brainstem [19] after a peripheral nerve lesion. However, the antigens, which are involved in the recruitment and the homing of activated T cells in the pathogenesis of NP, remain unknown.

Myelin basic protein (MBP) is the component of the compact myelin that is believed to participate in the maintenance of the major dense line and interactions of the myelin sheath with the cytosolic surfaces [20-23]. In the PNS, MBP comprises 5% to 15% of total myelin protein and is considered to be non-essential [22]. However, Th1-mediated autoimmune peripheral neuropathies in humans and the relevant experimental models induced by immunization of animals using immunodominant MBP and other myelin peptides are often painful [24]. It has been demonstrated that the autoimmune response to immunodominant MBP peptides assists in myelin clearance and regeneration after peripheral nerve injury [25]. Certain digest fragments of MBP and its splice variant (*Golli*-MBP) expressed in immune cells [26] are generated by matrix metalloproteinase (MMP) proteolysis and exhibit key T cell epitopes [27,28].

MMPs are a family of zinc-endopeptidases comprising collagenases, gelatinases, matrilysins, stromelysins, and membrane-type MMPs [29]. After peripheral nerve injury, gelatinases B (MMP-9) and A (MMP-2) degrade the blood-nerve barrier, release the pro-inflammatory cytokines, control immune cell infiltration and cell survival along the injured neural axis. These two MMPs are believed to consecutively initiate and maintain NP [5,30-34]. Having proposed that MMPs promote mechanical hypersensitivity via the proteolysis of myelin [5], we herein aimed to determine the specific mechanisms involved. Our present experimental evidence suggests that MMP-mediated fragmentation of MBP as a consequence of Wallerian degeneration exposes cryptic MBP epitopes, which are normally sheltered from immunosurveillance. These exposed immunodominant MBP peptide epitopes induce mechanical allodynia in both a T cell-dependent and -independent manner.

## Methods

### Reagents

Reagents were purchased from Sigma (St. Louis, MO) unless indicated otherwise. MBP2-18 (ASQKRPSQRSKYLATAS), MBP68-86 (AHYGSLPQKKSHGRTQDENP), MBP84-104 (ENPVVHFFKNIVTPRTPPPSQ), and scrambled MBP84-104 (NKPQTNVVEPFHRTFPIPPVS; sMBP84-104) peptides, derived from human MBP sequence (GenBank #AAH08749), were synthesized by GenScript. To prevent their degradation by exoproteinases, these 97% to 99% purity peptides were N- and C-terminally protected by acetylation and amidation, respectively. Because of the incomplete homology between the human and rodent MBP68-86 sequence, an additional Lys residue was inserted in the MBP68-86 sequence (underlined in the sequence above) to make the resulting peptide more uniformly applicable for our studies. Primers and *Taqman* oligonucleotide probes for rat MMP-9 (GenBank #NM_031055), GAPDH (GenBank #XO2231), and tumor necrosis factor-α (TNF-α, GenBank #NM_012675) were designed using Primer Express 2.0 software (Applied Biosystems) and obtained from Biosearch Technologies [30]. Similarly, the probes for rat IL-17A (GenBank #NM_001106897.1), were obtained from Applied Biosystems (Assay ID Rn01757168_m1). GM6001, a broad-spectrum MMP inhibitor, was purchased from Millipore. SB-3CT, a selective MMP-2/9 inhibitor, was purchased from EMD Biosciences. The following detection antibodies were used in our studies: rabbit anti-rat MMP-9 (Torrey Pines Biolabs, cat. #TP221, 1:500), goat anti-mouse MMP-9 (R&D Systems, cat. #AF909; 1:250), rabbit anti-S100 (Dako, cat. #Z0311, 1:500), murine anti-CD68 (clone ED1, Abcam, cat. #ab31630; 1:100), rabbit anti-von Willebrand factor (vWF, Abcam, cat. #ab6994, 1:1,000), rabbit anti-Iba1 (Wako, cat. #019-19741, 1:500), rat anti-MBP (Abcam, cat. #ab40390, 1:250), murine anti-human MBP (clone 22, AbD Serotec, cat. #MCA686S, 1:250), murine anti-MHC II (clone OX6, Abcam, cat. #Ab6403, 1:200), mouse anti-T cell receptor alpha/beta (TCR; AbD Serotec, cat. #MCA453G, 1:200), mouse β-actin antibody (Sigma, cat. #A53166, 1:30,000), goat anti-mouse conjugated with Alexa 594 (Molecular Probes, 1:500, red), or goat anti-rabbit conjugated with Alexa 488 (Molecular Probes, 1:500, green). The nuclei were stained with DAPI (Molecular Probes, 1:20,000, blue). We also used a rabbit anti-MBP antibody (Millipore, cat. #AB5864, 1:1,000) that recognizes degraded MBP and that was generated against the YGSLPQKSQRSQDENPVV MBP69-86 synthetic peptide (the guinea pig sequence) as an immunogen. Antibodies were diluted in TBS containing 0.1% Tween-20 and 1% normal goat serum.

### Animals, surgery, and therapy

All animals were housed at 22 °C under a 12 h light/dark cycle with food and water *ad libitum.* Animals were anesthetized with 4% isoflurane (Aerrane; Baxter) in 55% oxygen or a rodent anesthesia cocktail containing Nembutal (50 mg/mL; Abbott Labs) and diazepam (5 mg/mL) in 0.9% PBS (Steris Labs). Sprague–Dawley rats (*n* = 144, 200–225 g adult females), athymic nude rats (rnu^−/−^, Hsd:RH-*Foxn1*^*rnu*^*n* = 6, 8-week-old females) and their heterozygous controls (rnu^+/−^, Hsd:RH-*Foxn1*^*rnu*^*/Foxn1*^*+*^*n* = 6, 8-week-old females) were obtained from Harlan Labs. The common sciatic nerve was exposed unilaterally at the mid-thigh level. Four loosely constrictive chromic gut sutures were tied around the nerve to produce CCI [35]. SB-3CT (10 mg/kg body weight in 10% DMSO) was injected i.p. twice: first at the initiation of CCI and then in 24 h. Sol‐vent alone was used as a vehicle. In a separate group of animals, the exposed naïve sciatic nerves received an intraneural injection of an MBP peptide (50 μg) in 5 μL PBS, or an equal volume of PBS as a vehicle using a 33-gauge needle on a Hamilton syringe. For a sham-operated control, the sciatic nerve was exposed but otherwise not manipulated. The sciatic nerves and ipsilateral dorsal horn of the spinal cords were collected for analyses. FVB.Cg-Mmp9tm1Tvu/J (MMP-9^−/−^*n* = 6; 20 g, adult females) and wild-type FVB/NJ (WT, *n* = 6; 20 g, adult females) mice were obtained from Jackson Labs. The sciatic nerve was exposed unilaterally at the mid-thigh level and crushed using fine, smooth-surface forceps twice for 2 s each. The animals were sacrificed by an overdose of the Nembutal/diazepam cocktail, followed by Beuthanasia (100–150 mg/mL, i.p., Schering-Plough Animal Health). The animals were handled according to the NIH Guide for the Care and Use of Laboratory Animals and the required protocols were approved by the Institutional Animal Care and Use Committee.

### Two-dimensional liquid chromatography/tandem mass spectrometry/mass spectrometry (2D-LC/MS/MS), proteomics, and pathway analysis

The rat sciatic nerves were isolated, snap-frozen in liquid N_2_ and stored at −80 °C. The samples were homogenized, sonicated, extracted 60 min at ambient temperature in 100 mM Tris–HCl, pH 8.0, containing 8 M urea and the protease and phosphatase inhibitor cocktails, and the insoluble material was removed by centrifugation (16,000xg; 15 min). The supernatant samples (at least 0.5 mg total protein each) were then processed by the Proteomics Core facility of the Sanford-Burnham Medical Research Institute. The samples were reduced (10 mM tris(2-carboxyethyl) phosphine, 37 °C, 30 min), alkylated (20 mM iodoacetamide, 37 °C, 40 min in the dark), and digested using Modified Trypsin, Mass Spectrometry Grade (Promega; 1:100 w/w ratio; 37 °C, 16–18 h). The samples were desalted using a SepPack cartridge, dried using a SpeedVac and re-suspended in 0.1 mL 5% formic acid. The resulting peptides were separated into 24 fractions using an offline Michrom MDLC pump (Michrom) with a Michrom Strong Cation Exchange column. The 1/10 aliquot of each peptide fraction was analyzed using an LTQ-Orbitrap XL mass-spectrometer (Thermo Scientific) and a 15 cm Michrom Magic C18 column coupled with a low-flow Michrom ADVANCED device. The data were analyzed by Sorcerer Enterprise v.3.5 software (Sage-N Research) using the ipi.Rat.v3.56 protein database. 57 Da were added to cysteines to identify carboxyamidomethylated cysteines, 16 Da were added to methionines to identify oxidated methionines. The search results were sorted, filtered, and statistically analyzed using a trans-proteomic pipeline (TPP) (Institute for Systems Biology, Seattle, WA) with a 90% minimum probability score and an error rate ≤2%. An additional search was performed using a Prolucid search algorithm with a DTASelect function via an Integrated Proteomics Pipeline (IP2) server. Relative levels of the proteins in the samples were then analyzed using IP2 for a Label-Free differential peptide/protein analysis. The final data were subjected to bioinformatics analyses using Ingenuity IPA 8.7 software (Ingenuity Systems).

### Real-time qRT-PCR, genome-wide transcriptional profiling, and pathway analysis

The rat sciatic nerves were isolated and stored in RNA-later (Ambion) at −20 °C. Primers and Taqman probes were optimized to amplification efficiency of 100.1-100.3% [30]. Total RNA was extracted using TRIzol (Invitrogen) and purified on an RNeasy mini column (Qiagen). The RNA purity was estimated by measuring the OD260/280 and the OD260/230 ratios. The integrity of the RNA samples was validated using an Experion automated electrophoresis system (Bio-Rad). The samples were treated with RNase-free DNAse I (Qiagen). cDNA was synthesized using a SuperScript first-strand RT-PCR kit (Invitrogen). Gene expression levels were measured in a Mx4000™ Multiplex Quantitative PCR System (Agilent Technologies) using 50 ng of cDNA and 2x *Taqman* Universal PCR Master Mix (Ambion) with a one-step program: 95 °C, 10 min; 95 °C, 30 s; 60 °C, 1 min for 50 cycles. Duplicate samples without cDNA (a no template control) showed no contaminating DNA. Relative mRNA levels were quantified using the comparative delta Ct method [36] and glyceraldehyde 3-phosphate dehydrogenase (GAPDH) as a normalizer. The fold change between experimental and control samples was determined using the Mx4000 software.

For the genome-wide transcriptional profiling, the samples of total RNA (50 ng) from the wild-type and athymic nude rat nerves and spinal cord tissues were labeled using LowInput QuickAmp Labeling Kit and Cy3-CTP (Agilent Technologies). The labeled RNA samples were hybridized 17 h at 65 °C to SurePrint G3 Rat GE 8x60K slides (Agilent Technologies). Slides were scanned using an Agilent C Scanner. The raw data were processed using Feature Extraction software version 10.5. The initial analysis and normalization to the median were performed using GeneSpring GX software (Agilent). Differentially expressed mRNAs with signal intensities higher than two-fold over the background standard deviation were filtered by *t*-test. The statistically significant data only (*P* < 0.05) were used to calculate gene expression ratios in the samples. The gene expression data have been deposited to GEO database (accession # GSE34868, http://www.ncbi.nlm.nih.gov/geo/info/linking.html). The final data were analyzed using Ingenuity IPA 9.0 software.

### MMP-9 purification and proteolysis of MBP *in vitro*

The recombinant pro-form of MMP-9 was purified from the serum-free medium conditioned by the stably transfected HEK293 cells using the gelatin-column chromatography. The purity of the isolated MMP-9 samples was confirmed using SDS-PAGE in a 4-20% gradient acrylamide gel followed by Coomassie staining. Only the samples with purity over 95% were used in our studies. Purified pro-MMP-9 was activated using 4-aminophenylmercuric acetate. The concentration of the catalytically active MMP-9 was determined using a fluorescent assay by active site titration against a standard solution of a GM6001 of known concentration. (7-methoxycoumarin-4-yl) acetyl-Pro-Leu-Gly-Leu-(3-[2,4-dinitrophenyl]-L-2,3-diaminopropionyl)-Ala-Arg-NH_2_ (Bachem) was used as a fluorescent substrate [27,37].

Human MBP (4 μg; approximately 11 μM) was co-incubated with activated MMP-9 (1–100 nM; an enzyme-substrate ratio 1:100–1:10,000) in 50 mM HEPES, pH 6.8, supplemented with 10 mM CaCl_2_ and 50 μM ZnCl_2_, for 1 h at 37 °C. The total volume of the reactions was 20 μL. Where indicated, GM6001 (2.5 μM) was added to the reactions to inhibit MMP-9. The cleavage reaction was stopped using a 5xSDS sample buffer. The digest samples were analyzed by SDS-PAGE and by MALDI-TOF MS using an Autoflex II MALDI TOF/TOF instrument (Bruker Daltonics). For MS analysis, the reactions were cooled on ice and equal volumes (2 mL) of a sample and of a sinapic acid (20 mg/mL) in 50% acetonitrile-0.1% trifluoroacetic acid solution were mixed, spotted directly on a MALDI target plate, air-dried, and co-crystallized for 10 min. Mass spectra were processed with FlexAnalysis 2.4 software (Bruker Daltonics). The singly charged cleavage products, which were observed only in the cleavage reactions but not in the controls, were recorded and processed further.

### Gelatin zymography

Sciatic nerves were isolated, snap-frozen in liquid N_2_, and stored at −80 °C. Proteins were extracted in 50 mM Tris–HCl, pH 7.4, containing 1% Triton-x 100, 150 mM NaCl, 10% glycerol, 0.1% SDS. Extract aliquots (10–70 μg total protein as determined by BCA Protein Assay, Pierce) were analyzed using 10% acrylamide gels co-polymerized with 0.1% gelatin. After electrophoresis, gels were washed in 2% Triton X-100 for 30 to 60 min at ambient temperature, incubated for 16 to 18 h at 37 °C in 50 mM Tris–HCl buffer, pH 7.4, containing 10 mM CaCl_2_ and 1 μM ZnCl_2_ and 0.2 mM sodium azide, and stained with Coomassie Blue R250 to visualize the gelatinolytic activity bands.

### Neuropathology, immunohistochemistry, and microscopy

Plastic-embedded transverse nerve sections (0.75 μm each) were used for neuropathologic evaluation. Sciatic nerves were isolated and placed in 2.5% glutaraldehyde in 0.1 M phosphate buffer, osmicated, dehydrated, and embedded in araldite resin. Sections were cut with a glass knife on an automated Leica RM2065 microtome and stained using methylene blue Azure II. Immunohistochemistry was performed in tissues fixed in 4% para‐formaldehyde, embedded in paraffin, or cryoprotected in graded sucrose and embedded into OCT compound in dry ice. The 10-μm sections, when required, were deparaffinized using xylene and rehydrated in ethanol and PBS, immersed in 0.5% sodium borohydride followed by treatment with the antigen retrieval reagent (Dako) for 5 min at 95 °C, then for 20 min at ambient temperature. Teased nerve fibers were prepared from the transected and de-sheathed sciatic nerves. Nerve bundles were separated using a pair of fine smooth microforceps. Individual fibers were teased out using 0.20-0.22 mm acupuncture needles (Vinco, Oxford Medical Supplies) on a glass slide, dried at ambient temperature and stored at −20 °C. Non-specific binding was blocked using PBS containing 5% normal goat serum and 0.25% Triton X-100. The sections were incubated with a primary antibody (4 °C, 16–18 h) followed by an Alexa 488-conjugated (green) or Alexa 594-conjugated (red) species-specific secondary antibody (Invitrogen, 1 h, ambient temperature). The nuclei were stained with DAPI (5 min). Sections were mounted using a Slowfade Gold antifade reagent (Molecular Probes). The images were acquired using a Leica DMR microscope and Openlab 4.04 imaging software (Improvision).

### Behavior tests

Sensitivity to non-noxious mechanical stimuli was measured by von Frey testing [38]. Rats were acclimated to being on a suspended 6-mm wire grid. The plantar surface of the hindpaw was stimulated within the spinal nerve innervation area using calibrated von Frey filaments (Stoelting). Stimuli were applied for 4 to 6 s with a 0.4 to 15.0 g buckling force to the mid-paw plantar surface. In the event of a positive response, the next weaker stimulus was chosen for the next measurement. In the absence of a response, a stronger stimulus was presented. This consecutive way of applying filaments was continued until six responses in the immediate vicinity of the 50% threshold were obtained. The resulting sequence of positive and negative responses was used to interpolate the 50% withdrawal threshold, determined using the up-down method. Stimuli were separated by several seconds or until the animal was calm with both hind paws placed on the grid. Paw withdrawal latency to a thermal stimulus was measured by a Hargreaves testing device [39]. The hind paw was stimulated by a radiant heat source. Withdrawal of the paw from the heat source was measured four times to calculate the mean withdrawal latency. A maximal cut-off of 20 s was used to prevent tissue damage. The interval between two trials on the same paw was at least 5 min. Spontaneous pain-like behavior was measured as described in [40]. Each animal was placed in a 19 x 31 cm plexiglass cylinder and allowed to habituate. A 2 min testing period included continuous pressing of one of six (0–5) numerical keys on a computer keyboard, corresponding to the instantaneous behavior of the animal, rated by the positions of the injured hind paw as follows: 0, the paw is placed normally on the floor; 1, the paw is placed lightly on the floor and the toes are in a ventroflexed position; 2, only the internal edge of the paw is placed on the floor; 3, only the heel is placed on the floor and the hind paw is in an inverted position; 4, the whole paw is elevated; 5, the animal licks the paw. The measurements were repeated twice within 2 h. An index for noxious behavior was calculated by multiplying the time that rat spent in each behavior by a weighting factor for that behavior, and divided by the length of the observational period, using the formula: [0 t0 + 1 t1 + 2 t2 + 3 t3 + 4 t4 + 5 t5]/120 s, where t0-t5 are the time in sec spent in behaviors 0–5, respectively. The three values corresponding to three blocks of 120 s were averaged to determine the spontaneous pain score for each rat. All tests were performed daily for 3 days before peptide injection and then daily thereafter by an investigator blinded to the experimental groups.

### Data analyses

Statistical analyses were performed using KaleidaGraph 4.03 (Synergy Software) or SPSS 16.0 (SPSS) software by a two-tailed, unpaired Student’s *t*-test for comparing two groups, or analyses of variance (ANOVA) for repeated measures for comparing three or more groups, followed by the Tukey-Kramer post-hoc test, unless specified otherwise. *P* values ≤ 0.05 were considered significant.

## Results

### Proteomics identifies prominent Th cell signaling that follows nerve injury

**Figure 1:**
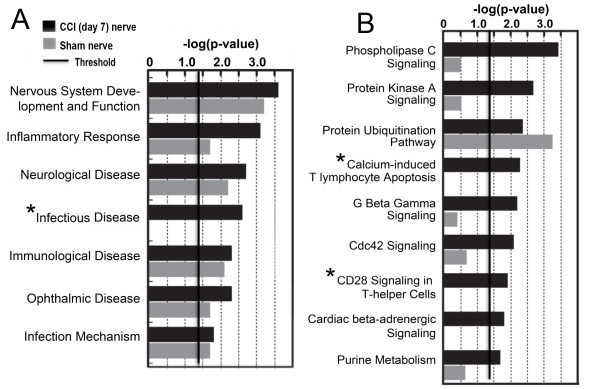
**T-helper cell signaling is induced after CCI.** Ingenuity pathway analysis of the canonical pathways (**A**) and signaling cascades (**B**) using the proteins unique to sciatic nerve proteome at day 7 after CCI or sham operation. The bars represent -log (*P* value) for a system or a pathway to be represented with a threshold set at 1.4 (*P* < 0.05) in *n* = 3/group. CCI presents as an infectious disease, with CD28 Th cell signaling and T cell apoptosis among unique pathways activated relative to sham-operated nerve (asterisks).

The importance of the unbiased screening of the neurobiological mechanisms contributing to neuropathic pain has been recently emphasized [41]. Specifically, proteomics analysis of the nerve using mass spectrometry has an advantage of simultaneously assaying the axonal, glial and immune cell proteins and peptides ultimately involved in the generation of action potentials in the course of the NP development [42]. Thus, mass spectrometry analysis of the rat sciatic nerve proteome at week 1 post-CCI, when T cells infiltrate the nerve [9-11,43], and control (sham-operated) nerves unambiguously identified 1,845 common individual proteins in normal and CCI nerves. A total of 320 and 441 additional individual proteins were detected only in the CCI and sham nerve samples, respectively (Additional file 1: Table S1 and Additional file 2: Table S2, respectively). Ingenuity software was used to analyze these results further and to identify the canonical signaling pathways, which were representative for our dataset. Thus, the infectious disease, the CD28 T-helper signaling, and the calcium-induced T cell apoptosis pathways are the major pathways that characterize the CCI proteome samples relative to the sham samples (Figure 1). Table 1 identifies the individual molecules that contribute to these T cell related pathways in CCI samples. According to Ingenuity, other up-regulated pathways in our CCI samples as compared with the sham-operated nerve samples also include inflammatory response, phospholipase C, and protein kinase A signaling pathways.

**Table 1:** List of molecules in T cell-related canonical pathways in CCI

**Biological function**	***P*value**	**Molecules**
Infectious disease	2.41E-03 to 4.67E-02	AFG3L2, AHCTF1, ALKBH3, BCLAF1, C1R, DDX23, DPM1, ETHE1, FLII, GANAB, HLA-DQB1, IMPDH1, MAPT, MERTK, NUP62, PPP3CA, PPP3CB, PYCRL, RAB6A, RANBP2, RPL10A (includes EG:4736), SFRS2, STXBP1, STXBP1, TIMM8A, TOP2B, TPPP, TPPP, USP39, ZMPSTE24
CD28 signaling in T-helper cells	1.25E-02	FYN, HLA-DQB1, PDPK1, PPP3CA, PPP3CB, PTPN6
Calcium-induced T lymphocyte apoptosis	3.87E-03	ATP2A3, HLA-DQB1, PPP3CA, PPP3CB, PRKCI

Ingenuity pathway analysis of the 2D-LC-MS/MS sciatic nerve proteome (day 7) after sham operation (normal) or CCI. *P* values for each biological function calculated by Fisher’s exact test are indicated.

These unbiased data highlighted the principal role of the infiltrating T cells in the nerve post-CCI and guided, at least partly, our follow-up research efforts. Having implicated MMP proteolysis of myelin in initiating mechanical allodynia [5], we hypothesized that the MMP-generated MBP digest peptides in the course of nerve injury comprise the cryptic T cell epitopes, which are sheltered from immunosurveillance in the intact nerve.

### Acute MMP-2/9 inhibition blocks CCI-induced allodynia and neuroinflammation

**Figure 2:**
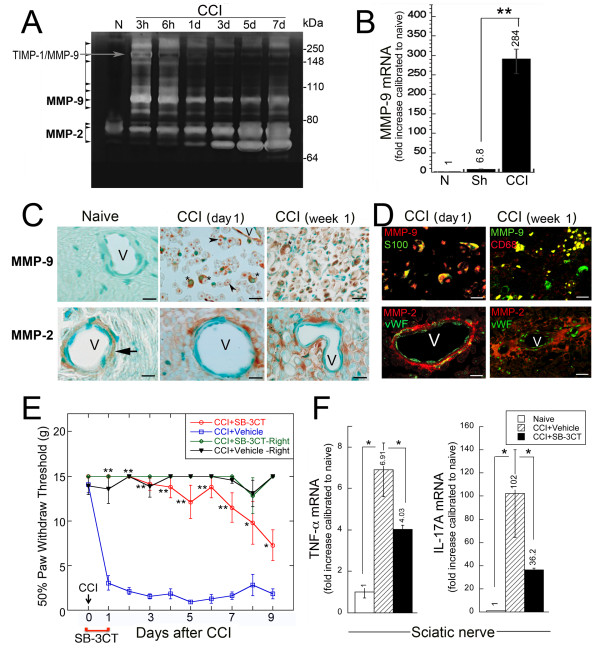
**Acute MMP-9/2 inhibition attenuates CCI-induced allodynia and neuroinflammation.** (**A**) Gelatin zymography of sciatic nerve extracts (70 μg total protein/lane) obtained at 3 h, 6 h, 1 day, 3 days, 5 days, and 7 days post-CCI. The arrows point to the MMP-9 and MMP-2 species. (**B**) *Taqman* qRT-PCR for MMP-9 in the sham (Sh) and CCI sciatic nerves (day 1). The mean relative mRNA ± SEM of *n* = 6/group normalized to GAPDH compared to naïve (N) nerve (**, *P* < 0.01). (**C**) Immunostaining of MMPs (2’2-diaminobenzidine, brown) in the sciatic nerve at day 1 and week 1 post-CCI. Methylene Blue, counterstain. MMP-9 is in the Schwann cells (asterisks) and axoplasm (arrowheads). MMP-2 is at the blood-nerve barrier (arrow), surrounding the vessel (V) endothelium, perivascular areas and at the Schwann cell plasma and basement membranes. Representative of *n* = 3/group. Scale bar, 40 μm. (**D**) Top panel, MMP-9 (red, left; green, right) co-localizes (yellow) with the S100-positive Schwann cells (left) at day 1 and with the CD68-positive macrophages (right) at week 1 post-CCI. Bottom panel, MMP-2 (red) localizes along the blood-nerve barrier and vWF-positive endothelial cells (green) at both time-points. Representative of *n* = 3/group. Scale bar, 40 μm. (**E**) von Frey testing after CCI and administration of SB-3CT (10 mg/kg) or the vehicle (10% DMSO) i.p., twice, at days 0 and 1 post-CCI. Decline in the withdrawal threshold in the ipsilateral (ipsi) to CCI hind paw corresponds to allodynia. SB-3CT-treated rats displayed no sensitivity to stimuli below 10–15 grams, comparable to that of contralateral (contra), uninjured hind paws. The mean withdrawal threshold (gram force; g) ± SEM of *n* = 6/group (**, *P* < 0.01; *, *P* < 0.05). (**F**) *Taqman* qRT-PCR for TNF-α and IL-17A analyzed at the completion of E (day 9 post-CCI). The mean relative mRNA ± SEM of *n* = 5/group normalized to GAPDH compared to the contralateral nerve (*, *P* < 0.05).

Broad-spectrum MMP inhibition suppressed immune cell infiltration after nerve injury [5]. Within week 1 post-CCI, when T cells infiltrate the nerve, MMP-2 and MMP-9 activity was differentially induced in the nerve (Figure 2A). MMP-9 was undetectable in the naïve nerve but it was sharply up-regulated following CCI. MMP-9 appeared as a prominent 92 kDa latent pro-enzyme, an active 88 kDa monomer and high molecular weight homo- and heterodimers at 3 h after CCI. A heterodimer of MMP-9 with a tissue inhibitor of metalloproteinases (TIMP)-1, found in nerve within day 1 post-injury [44], partially protected gelatin from degradation (Figure 2A). Constitutive expression of the 72 kDa MMP-2 latent proenzyme was observed in the naïve nerve. By day 1, MMP-2 was partially activated, resulting in a 68 kDa enzyme band. We quantified the increase in the MMP-9 expression at day 1 post-CCI. MMP-9 mRNA levels increased 284-fold and 42-fold compared with the naïve and sham-operated nerve samples, respectively (Figure 2B). According to our immmunostaining studies, the crescent-shaped Schwann cells and the vessel endothelial cells expressed MMP-9 at this time-point (Figure 2C). The MMP-2 immunoreactivity was detected in the perivascular areas, and also on the Schwann cell plasma and/or basement membranes. The MMP-9 immunoreactivity was also observed in the axoplasm of myelinated fibers in nerve post-injury [44]. Schwann cells (identified by S100) and macrophages (identified by CD68) were the main source of MMP-9 in injured nerve at day 1 and week 1 post-CCI, respectively (Figure 2D). Vessel endothelial cells (identified by vWF) produced MMP-2 at both time-points. These MMP expression patterns are consistent with the previous reports [45,46].

Ongoing therapy using the broad-spectrum MMP or selective MMP-2/9 inhibitors suppressed the development of NP [5,33]. To test if the acute selective MMP-9/2 inhibition suppressed the CCI-induced pain, we employed SB-3CT. SB-3CT is a selective, mechanism-based MMP-2/9 inhibitor (k_i_ = 14-600 nM) [47] shown efficacious in promoting the functional recovery from brain and spinal cord injury [48,49]. SB-3CT (10 mg/kg, i.p.) was administered twice, during the CCI operation and then in 24 h. A significant and stable drop in the mechanical withdrawal threshold (severe allodynia) was evident readily after CCI in the vehicle-treated animals (Figure 2E). In contrast, acute SB-3CT therapy maintained the high threshold levels for up to 9 days in the animals. As measured at the end of the behavioral tests (day 9 post-CCI), acute SB-3CT therapy decreased the levels of the pro-nociceptive TNF-α and IL-17A in the CCI samples compared to the vehicle group (Figure 2F). There was no significant change in the corresponding lumbar 4/5 DRG and spinal cord expression of either TNF-α or IL-17A at this time-point (data not shown). Thus, immediate and acute inhibition of MMP-9/2 proteolysis prevented the CCI-induced mechanical allodynia and neuroinflammation for longer than week 1 of injury.

### MMP-9 degrades MBP *in vitro* and *in vivo*

**Figure 3:**
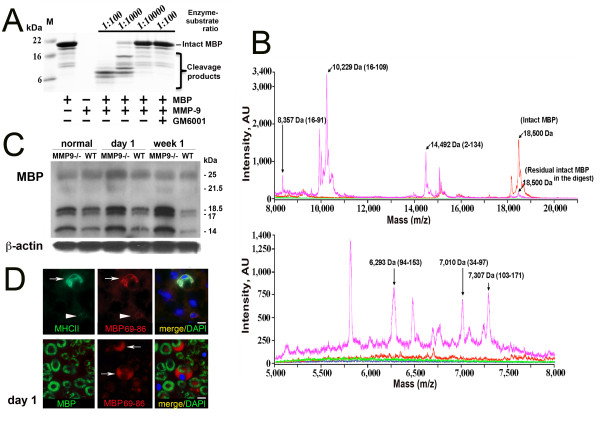
**MMP-9 proteolysis yields MBP epitopes.** (**A**) *In vitro* cleavage of MBP (18 kDa) by MMP-9. Human MBP (4 μg; 11 μM) was co-incubated with MMP-9 at an enzyme-substrate ratio of 1:100–1:10,000 in 50 mM HEPES, pH 6.8, 10 mM CaCl_2_ and 50 μM ZnCl_2_ for 60 min at 37 °C. Where indicated, GM6001 was added to the reactions. The digests were analyzed using SDS-PAGE. M, molecular weight markers. (**B**) Representative MALDI-TOF MS spectra of the MBP samples. MBP (11 μM) was co-incubated for 60 min at 37 °C with MMP-9 (10 nM). The digests were analyzed using a Bruker Daltonics Autoflex II MALDI TOF mass spectrometer to determine the molecular mass of the resulting peptides. The high and low molecular mass fragments are shown in the top and bottom panels, respectively. The spectra represent arbitrary units (AU) for the MBP digest (magenta), the intact MBP (red), and the buffer alone (green). The numbers in parentheses show the numbering of the peptide in the MBP sequence. (**C**) Western blotting for MBP in the sciatic nerves at 1 day and week 1 post-crush or contralateral (normal) nerves in MMP-9 null (^−/−^) or a wild-type (WT) mice (pooled from *n* = 3 mice/lane). β-actin, protein loading control. (**D**) Immunostaining of MBP69-86 using a specific antibody (AB5864, Millipore, red) and MHCII (green, top) or intact MBP (green, bottom) in the myelinating Schwann cells (arrows) and other cells (arrowheads) at day 1 post-CCI. DAPI, blue. Representative of *n* = 3/group. Scale bar, 10 μm.

Next, we tested if MBP cleavage by MMP-9 uncovered cryptic T cell epitopes normally sheltered from immunosurveillance.A noticeable level of MMP-9 proteolysis of human MBP (18.5 kDa) was observed *in vitro* in 1 h at an enzyme-substrate molar ratio as low as 1:10,000, while the degradation of MBP was largely accomplished at a 1:1,000 molar ratio (Figure 3A). GM6001, a broad-spectrum MMP inhibitor, abolished MMP-9 proteolysis of MBP. The mass of the MBP digest fragments and, consecutively, their peptide sequence was determined by MALDI-TOF MS (Figure 3B; Table 2). The MBP digest fragments comprised the known immunogenic sequence regions of MBP, MBP84-104, and MBP68-86 sequences, capable of causing experimental autoimmune encephalomyelitis or neuritis in animal models [50-54].

**Table 2:** **MMP-9-digested MBP peptides*in vitro***

**ASQKRPSQR^10^ HGSKY↓LATAS^20^ TMDHARHGFL^30^ PRH↓RDTGILD^40^ SIGRFFGGDR^50^ GAPKRGSGKD^60^ SHHPARTAHY^70^ GSLPQKSHGR^80^ TQDENPVVHF^90^ F↓KN↓IVTP↓RTP^100^ PP↓SQGKGRGL^110^ SLSRFSWGAE^120^ GQRPGFGYGG^130^ RASD↓YKSAHK^140^ GFKGVDAQGT^150^ LSK↓IFKLGGR^160^ DSRSGSPMAR^170^ R^171^**		
Peptide sequences	Molecular mass, Da	
	Calculated	Measured
A^2^SQKRPSQRHGSKYLATASTMDHARHGFLPRHRDTGILDSIGRFFGGD	14,482	14,498
RGAPKRGSGKDSHHPARTAHYGSLPQKSHGRTQDENPVVHFFKNIVTP		
RTPPPSQGKGRGLSLSRFSWGAEGQRPGFGYGGRASD^134^		
L^16^ATASTMDHARHGFLPRHRDTGILDSIGRFFGGDRGAPKRGSGKDSH	10,229	10,247
HPARTAHYGSLPQKSHGRTQDENPVVHFFKNIVTPRTPPPSQGKGRG^109^		
L^16^ATASTMDHARHGFLPRHRDTGILDSIGRFFGGDRGAPKRGSGKDSH	8,357	8,356
HPARTAHYGSLPQKSHGRTQDENPVVHFF^91^		
S^103^QGKGRGLSLSRFSWGAEGQRPGFGYGGRASDYKSAHKGFKGVDA	7,307	7,296
QGTLSKIFKLGGRDSRSGSPMARR^171^		
R^34^DTGILDSIGRFFGGDRGAPKRGSGKDSHHPARTAHYGSLPQKSH	7,010	7,018
GRTQDENPVVHFFKNIVTP^97^		
I^94^VTPRTPPPSQGKGRGLSLSRFSWGAEGQRPGFGYGGRASDYKSAHK	6,293	6,284
GFKGVDAQGTLSK^153^		

MMP-9 proteolysis of human MBP was performed as detailed in Figure 3, followed by MALTI-TOF MS analysis of the digest fragments. The proteolysis of MBP by MMP-9 was performed at a 1:1,000 enzyme-substrate ratio. The sequences of intact MBP and digest fragments are shown. The arrows indicate the MMP-9 cleavage sites. The dot indicates the initiating Met-1.

In the PNS, MBP is presented as several splice variants (14, 17, 18.5, and 21.5 kDa) and charge isoforms with the potentially different posttranslational modifications [22,23]. We have previously demonstrated that the nerves of MMP-9 null mice accumulated different MBP isoforms both early (day 1) and later (day 10) post-injury [5,32]. Consistent with our previous data and the ability of MMP-9 to proteolyze 18.5 kDa MBP *in vitro*, the 18.5 kDa MBP and also the low molecular weight MBP species accumulated in the MMP-9-deficient nerves within 1 day post-injury (Figure 3C). At day 1 post-injury, MMP-9 co-localized with the degraded MBP in myelinating Schwann cells in the wild-type animals [5], as detected using a specific AB5864 antibody against degraded myelin.

### The endogenous MBP epitopes in Schwann cells, monocytes, and A-fibers of the injured nerve

Because monocytes infiltrate the nerve only after day 2 post-injury, we aimed to determine the cells involved in MBP degradation and antigen presentation at day 1 post-CCI. The AB5864 antibody to the degraded MBP we used was generated against the MBP69-86 peptide as an immunogen. As a result, in the areas of demyelination, this antibody efficiently recognized the MBP69-86 cryptic epitope from the central portion of MBP rather than the full-length intact MBP in which this epitope was sheltered [55]. Non-lesioned nerves were not reactive with the AB5864 antibody [5], and the immunoreactivity patterns of MBP69-86 and intact MBP were clearly distinct in the injured nerve (Figure 3D). Note that myelinating Schwann cells present as crescent structures due to their association with myelinated axons (reactive for intact MBP) at a 1:1 ratio. At day 1 post-CCI, the endogenous MBP69-86 epitope was detected in MHCII-reactive Schwann cells as well as in certain other cell types (Figure 3D).

**Figure 4:**
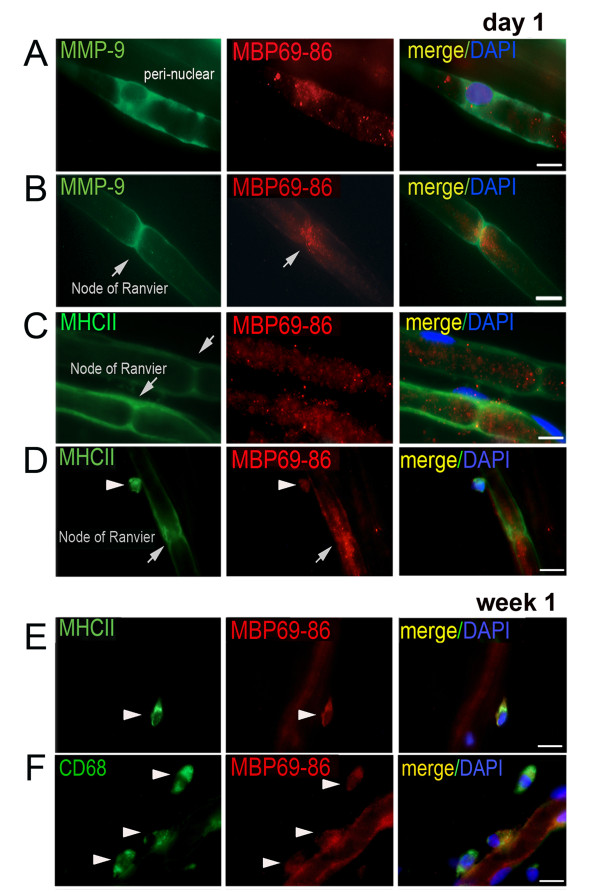
**Accumulation of MBP69-86 at the nodes/paranodes of myelinated fibers.** Dual-immunostaining of MBP69-86 using a specific antibody (AB5864, Millipore, red, A-F) and MMP-9 (green, A-B), MHCII (green, C-E) or CD68 (green, F) in teased nerve fibers at day 1 (A-D) or week 1 (E-F) after and immediately proximal to transection. DAPI, blue. MMP-9 and MBP69-86 co-localize in the perinuclear and cytosolic regions of the myelinating Schwann cells (**A**). MMP-9, MBP69-86 and MHCII co-localize in the paranodal/nodal areas of presumably, A-fibers (arrows, **B**, **C**, **D**). Additionally, MBP69-86 is found in small, MHCII-positive cells adjacent to the fibers (**E**, arrowhead), such as CD68-reactive macrophages (**F**, arrowheads). Representative of approximately 20 individual fibers in *n* = 3/group. Scale bars, 10 μm.

The distribution of endogenous MBP69-86, MHCII and MMP-9 within the domains of myelinated fibers was analyzed in the teased rat sciatic nerve preparations at day 1 and week 1 after transection (Figure 4). Due to Wallerian degeneration altering the integrity of A-fibers in the distal segment, we analyzed the fibers from the segment immediately proximal to transection, the site where MMP-9 expression and activity were induced [44,56]. MMP-9 co-localized with MBP69-86 in the Schwann cell cytoplasm and perinuclear areas of the teased nerve fiber preparations (Figure 4A). Intriguingly, at day 1 post-injury, MBP69-86 localized in the paranodal/nodal regions, in close proximity to MMP-9 (Figure 4B) and MHCII (Figure 4C) on the Schwann cell plasma and/or basement membranes. MHCII-positive round small cells adjacent to the myelinated fibers were reactive for MBP69-86 at day 1 (Figure 4D) and, especially week 1 (Figure 4E) post-injury. A number of MBP69-86-reactive macrophages (identified by CD68) were adjacent to the fibers (Figure 4F). The later finding may represent phagocytosed myelin in hematogenous or resident macrophages, or the degraded *Golli-*MBP, expressed in immune cells [26].

### MBP peptides induce allodynia

**Figure 5:**
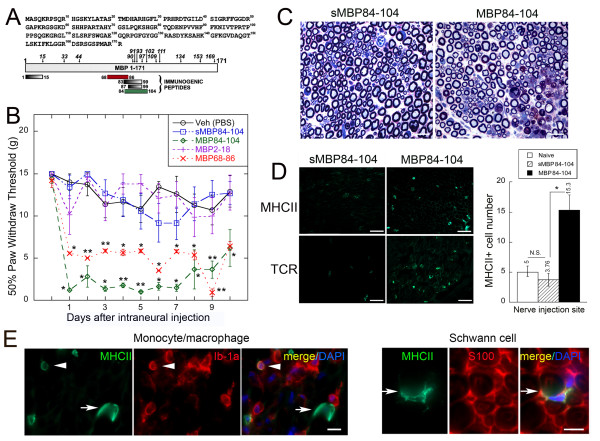
**Pro-nociceptive activity of MBP peptides.** (**A**) The 1–171 sequence of human MBP (GenBank #AAH08749). The immunogenic regions are shown at the bottom of the panel using human MBP residue numbering. Following MBP cleavage by MMP-9, the mass and, consecutively, the sequence of the digest fragments was determined by MALDI-TOF MS. The italicized numbers indicate the positions of the cleavage sites. (**B**) von Frey testing after the intraneural injection of MBP peptides (50 μg in 5 μL) or vehicle (PBS) into a naïve rat sciatic nerve. Within 1 day a decline in mechanical withdrawal thresholds was observed after the MBP84-104 and MBP68-86 injection. Control MBP2-18 and MBP84-104 scrambled (sMBP84-104) peptide induced no significant change in thresholds compared to PBS. The mean withdrawal thresholds (gram force; g) ± SEM of *n* = 6/group (**, *P* < 0.01, *; *P* < 0.05). (**C**) Methylene Blue Azure II staining in 1-μm-thick sciatic nerve sections. MBP84-104 produced myelin splitting and cell infiltration 3 days post-injection into the naïve nerve. Uncompromised axons, surrounded by a compact rim of myelin are observed in the nerves after the sMBP84-104 injection. Representative micrographs of *n* = 4/group. Scale bars, 20 μm. (**D**) Immunostaining of MHCII (green) or T cell receptor (TCR, green) in the nerve at 3 days after the MBP injection into the intact nerve. Scale bars, 25 μm. The graph represents morphometry of the mean MHCII-positive cell numbers in the sciatic nerves ± SEM of *n* = 4/group and three sections per *n* (*, *P* < 0.05). (**E**) Immunostaining of MHCII (green) and Iba1 in the monocytes/macrophages (red) or S100 in the Schwann cells (red) in the nerve after the MBP84-104 injection in the wild-type rats. Macrophages (arrowheads) and Schwann cells (arrows) represent MHCII-reactive cells in the nerve, exposed to the immunodominant MBP84-104 peptide. DAPI, blue. Representative micrographs of *n* = 4/group. Scale bars, 10 μm.

Our data indicate that as a result of MMP-9 proteolysis of MBP *in vitro,* cryptic epitopes are released at the N-terminal and central portions of the MBP sequence, including MBP68-86, MBP84-104 (summarized in Figure 5A). In agreement, the immunodominant MBP69-86 peptide sequence was produced in the injured nerve. Next, we analyzed the effect of the synthetic MBP peptides on pain-like behaviors. Mechanical and heat hypersensitivity and spontaneous pain-like behavior parameters were assessed in rats after a single intraneural injection of MBP84-104, MBP68-86, MBP2-18 and scrambled MBP84-104 (sMBP84-104) peptides (>97-99% pure, 50 μg each) into a naïve sciatic nerve (Figure 5B). A drop in the mechanical withdrawal threshold after the injection of the MB-P84-104 and MBP68-86 peptides corresponded to robust allodynia lasting for up to 9 days. In contrast, injection of MBP2-18 and sMBP84-104 was without effect and resulted in a withdrawal threshold comparable to PBS injection. Robust decline in the mechanical withdrawal threshold was also readily observed after a single injection of 10 μg of MBP68-86 or MBP84-104 (data not shown).

Further investigation was done using MBP-84-104 (the most potent modulator of allodynia) and its scrambled peptide control. The neuropathology of the respective nerves at the injection sites was analyzed at day 3 after the MBP84-104 or sMBP84-104 injection, when the difference in pain-like behavior was highly significant. In contrast with sMBP84-104, MBP84-104 produced focal myelin splitting, endoneurial edema and infiltration of phagocytic immune cells (Figure 5C). These findings were accompanied by an increase in the number of T cell receptor (TCR) and MHCII-reactive cells at the MBP84-10 injection site compared with the sMBP84-104 group (Figure 5D). Monocytes/macrophages (identified by Iba1) and Schwann cells (identified by S100) expressed MHCII after the MBP84-104 injection (Figure 5E). The effects of the MBP84-104 injection on the activation of the inflammatory pathways in the naïve nerve were analyzed further using the genome-wide gene expression profiling and bioinformatics analyses.

### T cell deficiency diminishes the pro-nociceptive MBP84-104 action

**Figure 6:**
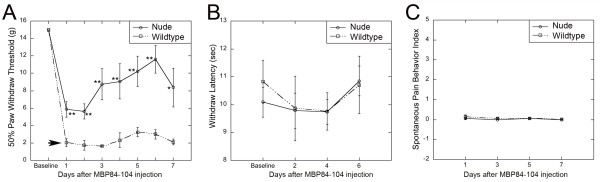
**MBP-induced allodynia is T cell-dependent**. (**A**) von Frey testing for mechanical allodynia in athymic nude (rnu^−/−^) and wild-type heterozygous (rnu^+/−^) rats after the MBP84-104 injection (50 μg in 5 μL) into the naïve sciatic nerve. The mean withdrawal thresholds (gram force, g) ± SEM of *n* = 6/group decline rapidly after the MBP84-104 injection into normal rats, corresponding to allodynia (arrow), but remain significantly higher in nude rats (**, *P* < 0.01; *, *P* < 0.05). (**B**) Hargreaves testing for thermal sensitivity. The mean paw withdrawal latency ± SEM of *n* = 6/group after thermal stimulation (radiant heat) before (baseline) and at the indicated days after the MBP84-104 injection were not different between the groups. (**C**) Spontaneous pain scoring of the MBP84-104 injected paw positioning for 6 min (3 x 120 s) using a 0–5 numerical scale demonstrates the absence of spontaneous pain-like behaviors in both rat types. The mean score ± SEM of *n* = 6/group.

**Figure 7:**
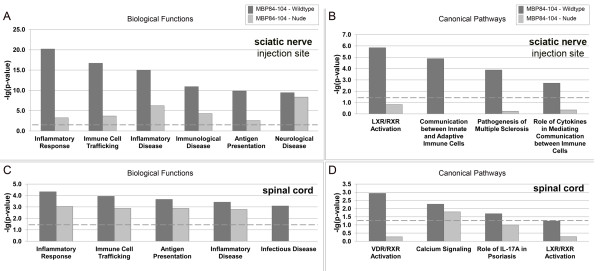
**Functional analysis of global gene expression in nerves and spinal cords after intraneural MBP84-104 injection.** Ingenuity pathway analysis of the gene expression used as the input list for generation of the top functional categories and canonical pathways (X-axis). The bars represent -log10 (*P* value) for a function or a pathway to be represented with a threshold (dashed line) set at 1.3 (*P* <0.05) in *n* = 6/group (right-tailed Fisher's exact test). Biological functions and canonical pathways are listed from most to least significant in the experimental MBP-injected animals in the injected compared to control contralateral sciatic nerves (**A**, **B**) or in the dorsal horns of the corresponding lumbar compared to control thoracic spinal cords (**C**, **D**) of the same animals. LXR, liver X receptor; RXR, retinoid X receptor; VDR, vitamin D receptor.

Athymic nude (rnu^−/−^) rats exhibit depleted T-cell populations in thymus-dependent areas of peripheral lymphoid organs. Because rnu^−/−^ rats are less susceptible to the development of NP [9], we used this strain to test our hypothesis that the pro-nociceptive effect of MBP84-104 depended on T cell production, infiltration or homing to the nerve and the corresponding spinal cord (Figures 6 and 7).

The mechanical withdrawal thresholds readily declined following a single MBP84-104 injection in the wild-type (rnu^+/−^) rats (Figure 6A). In turn, incomplete attenuation of allodynia was observed in nude rats. The threshold levels significantly increased by day 3 post-injection, although remained below the baseline. The injection of MBP84-104 did not cause a change in thermal withdrawal latencies (Figure 6B) or spontaneous pain-like behaviors (Figure 6C) in both rat types. We concluded that immunodominant MBP peptides, specifically MBP84-104, initiated mechanical but not thermal hypersensitivity in naïve animals, and that the pro-nociceptive activity of MBP84-104 was, at least partly, T cell-dependent.

At the completion of behavioral analyses (1 week after the MBP84-104 injection), the sciatic nerve (the injected and the contralateral side) and the dorsal horn spinal cord (the lumbar enlargement and above-the-level thoracic segments) from the wild-type and nude rats were collected for the further genome-wide transcriptional profiling. The aim of these experiments was two-fold: (1) correlate the decline in MBP84-104-induced allodynia with the decline in T cell response via the unbiased screening; and (2) identify T cell-independent changes induced by MBP84-104 that might persist longer in the T cell-deficient tissues. The top biological functions and pathways induced by the intraneural MBP84-104 injection both in the nerve and the cord were categorized as canonical inflammatory response, immune cell trafficking, inflammatory disease and antigen presentation pathways (Figure 7 and Table 3). In the nerve, 1 week after the MBP-84-10 injection the antigen presentation function was elevated approximately 10-fold (Figure 7A), communication signaling between innate and adaptive immune cells signaling was elevated five-fold (Figure 7B) and the T cell activation pathway was elevated 7.8-fold (Table 3), as compared to contralateral nerve in the same animals. In lumbar dorsal horn spinal cord, the intraneural MBP84-104 injection produced an over three-fold increase in antigen presentation function (Figure 7C), an about 1.8-fold increase in IL-17 signaling (Figure 7D) and 2.6-fold increase in T cell activation pathway (Table 3) relative to the thoracic segment in the same animals. The signaling cascades representative of autoimmune demyelination were activated at the MBP84-104 nerve injection site of the wild-type but not the nude rats (Figure 7B, Additional file 3: Figure S1). In addition, MBP84-104 activates calcium, liver X receptor (LXR), retinoid X receptor (RXR), and vitamin D receptor (VDR) signaling in the nerve and the corresponding spinal cord (Figure 7).

**Table 3:** MBP84-104 activates inflammatory pathways in nerve

Functions annotation	Nerve, -log (*P* value)		Spinal cord, -log (*P* value)	
	WT	Nude	WT	Nude
Immune response	20.2	-	4.32	-
Inflammatory response	15.8	-	2.79	1.42
Activation of T lymphocytes	7.8	-	2.64	-
Chemotaxis/aggregation of APCs	5.5	2.39	2.54	2.88
Chemotaxis of monocytes	4.2	2.61	1.86	2.36

Ingenuity pathway analysis of the genome 1 week after the MBP84-104 injection into the intact sciatic nerve in the wild-type and nude rats. Up-regulated functions as a result of inflammatory response in the injected nerve compared to the contralateral sciatic nerve and in the lumbar compared to the thoracic spinal cord, dorsal horn, are shown. Cutoff is set at *P* < 0.05 (−log10 > 1.3). Minus indicates that the Ingenuity software did not detect the presence of the pathway in the sample.

## Discussion

Definite progress in elucidating the immunological mechanisms of NP has been achieved in recent years [17,57-60]. However, the molecular and cellular processes that cause myelinated Aβ-afferents to enter nociceptive circuits after nerve damage remain poorly understood as yet [1,61]. There is growing evidence for the direct relationship between axonal demyelination and pain [2-4,6]. We have implicated MMP proteolysis of myelin, specifically MBP, in mechanical hypersensitivity [5]. Herein, we provide strong evidence that the MMP-generated MBP digest peptides comprising the potent immunogenic epitopes are released during Wallerian degeneration. We also demonstrated that these immunogenic MBP peptides initiate mechanical allodynia in both T cell-dependent and -independent manners.

### Multiphasic roles of MMPs in MBP/golli-MBP cleavage and NP

MMPs are key degrading proteases of MBP. The peptides generated because of MMP-9 proteolysis of various MBP/Golli-MBP isoforms include MBP84-104, 69–86, as well as other immunodominant regions [27,28,37,62,63]. At least six distinct peptides in the 6–14.5 kDa range are formed as a result of MMP-9 proteolysis of pure human 18.5 kDa MBP *in vitro*. Accordingly, an 18.5 kDa and other MBP isoforms accumulated in the MMP-9-deficient nerves. Further, more in-depth studies are required to assess the *in vivo* kinetics of MMP-9 proteolysis of multiple MBP/Golli-MBP sequence and post-translationally modified isoforms [23] over the course of nerve injury. The ability of MMP-9 to affect the MBP expression by regulation of Schwann cell signaling [56] also needs to be taken into consideration.

MMP-9 co-localizes with the endogenous MBP69-86 in the myelinating Schwann cell cytoplasm [5] and perinuclear areas, and in the paranodal/nodal regions of myelinated fibers. In addition, macrophages deposit MBP69-86 in the nerve. Among the *Golli*-MBP splice forms produced by immune cells, BG21 and J37 contain the cryptic epitope sequences [21,64-66] that we have found to be released by MMPs [27,37]. Because of the cleavage redundancy among the MMP family, several MMPs are likely to proteolyze MBP/*Golli*-MBP over the course of nerve injury. The differential ability of MBP84-104, 68–86, and 2–18 peptides to produce allodynia suggests that proteolysis of MBP influences susceptibility to NP.

In addition to protecting myelin from proteolysis, ongoing broad-spectrum MMP inhibition may prevent allodynia by protecting DRG neurons from apoptosis [5] and suppressing the expression of certain voltage-gated sodium channels [44], although the latter effect may also relate to the myelin integrity [2,4]. MMP-2/9 also release the pro-nociceptive cytokines (TNF-α and IL-1β) from their transmembrane precursors, promoting peripheral and spinal glial activation and immune cell-mediated pain [33,34,58]. The nerves acutely treated immediately post-CCI with the MMP-9/2 inhibitor, SB-3CT, sustain the low levels of the TNF-α expression (produced by Schwann cells, macrophages, endothelial, Th1, and other cells) and the IL-17A expression (produced by Th17 and other cells) [11,15,67]. Because MMP-9 and not MMP-2 is induced immediately post-injury and has been implicated in the initiation of NP [5,33], we attribute the effects of acute SB-3CT therapy mainly to MMP-9 inhibition. Likewise, therapy with TIMP-1 prevents NP by MMP-9 inhibition [33], as TIMP-1 binds MMP-9 stoichiometrically and blocks access of its catalytic site to substrates [29].

### T cells in pain and pro-nociceptive action of MBP

T cell-deficient animals are less susceptible to NP [9,11,12,14]. Progress has been made in identifying T cell subset phenotypes involved in NP. For example, a decline in hypersensitivity in CD4 null mice in the spinal nerve ligation model was resumed with adoptive transfer of CD4+ Th cells [12]. Specifically, transfer of Th1 cells (that produce pro-inflammatory cytokines) restores allodynia, as Th2 cells (that produce anti-inflammatory cytokines) sustain resistance to CCI-induced pain in nude rats, strongly implicating Th1 but not Th2 cells in promoting NP [9]. IL-17A, produced by a unique subset of Th17 cells, is detected at week 1 post-CCI [11], and IL-17 deletion protects from the development of NP [15].

Activated T cells patrol the intact PNS during immunosurveillance irrespective of their antigen specificity [31]. They infiltrate the nerve at 1 week after a physical nerve damage [9-11,43] via the coordinated action of chemokines, cytokines, and MMPs that compromise the blood-nerve barrier and trigger demyelination [31,68,69]. T cell infiltration into the spinal cord also contributes to the development of peripheral NP [13,14,16]. It is plausible that repeated exposure of MBP/*Golli*-MBP epitopes results in the formation of MBP-specific T cell clones, which then infiltrate the corresponding central segments, where antigen-presenting systems are in place [14,16,19]. It is interesting to point out that the classic MBP and *Golli-*MBP differentially regulate T cell signaling [70].

The ability of MBP84-104 to initiate allodynia is diminished in nude rats, indicating the presence of the T cell-dependent mechanism of MBP action in neuropathic pain. In agreement, our data clearly demonstrated that following the MBP84-104 injection there was no increase in the inflammation and antigen presentation signaling in both the nerve and the corresponding spinal cord in nude animals*.* However, both the ability of MBP84-104 to initiate pain shortly after the injection and residual hypersensitivity in the nude rats imply that there is an additional, T cell-independent component in the ability of MBP84-104 to promote pain. For example, MBP regulates intracellular calcium flow [71,72], a key factor in pain facilitation [73]. It appears that MBP84-104 (in a T cell-independent manner) affects calcium flow in the wild-type and nude rats.

### 1Schwann cells in myelin clearance and antigen presentation

MBP84-104 injection into the intact nerve induced MHCII in the macrophages and Schwann cells. The endogenous cryptic MBP69-86 epitope was detected in MHCII-reactive myelinating Schwann cells within day 1 post-injury. This is not surprising, since during the first few days post-injury, Schwann cells are actively involved in the degradation and removal of the myelin debris and in the presenting of myelin antigens [69,74-78]. The deposits of the endogenous MBP69-86 in CD68-reactive injured nerve may represent phagocytosed myelin debris or the *Golli* species, expressed by monocytes and other blood cells [26]. Overall, myelin degradation and clearance in the damaged PNS appears to consist of an early phase mediated by Schwann cells and resident macrophages [31] and an antibody-dependent later phase mediated by hematogenous macrophages [25,69]. Each phase of this event may have distinct function in the NP state.

### Neuro-immune interactions at A-fibers: mechanical *vs.* thermal hypersensitivity

Accumulation of MBP69-86 and MHCII around the nodes of Ranvier is intriguing. We speculate that MBP69-86 both activates the pro-nociceptive changes in the calcium flow [71,72] and facilitates the T cell homing at these action-generating sites on A-afferents. In agreement, MBP84-104 induced mechanical allodynia but not thermal hyperalgesia, at least in the intact nerve environment, and T cell-deficient IL-17 null mice develop resistance to mechanical allodynia but not thermal hyperalgesia [15]. There is a growing appreciation that differential mechanisms underlie these NP states, as A-afferents mediate mechanical allodynia and heat-nociceptive C-fibers mediate thermal hyperalgesia [79,80]. The MBP degradation and T cell homing to the regions which are immediately proximal to transection and in which elevated MMP-9 levels and other features of Schwann cell activation manifest [44,56,81], may explain pain facilitation despite the fiber loss at the lesion site. Finally, the present study does not distinguish between the myelinated afferents and efferents, supporting a model that demyelinating motor efferents contribute to nociceptive pain [3].

## Conclusions

The present data implicate proteolyzed MBP in pain. Over the course of Wallerian degeneration, the repeated exposure of the MBP epitopes normally sheltered from immunosurveillance may lead to the formation of the MBP-specific T cell clones and a self-sustaining immune reaction both of which contribute to the transition of ‘protective autoimmunity’ and acute pain to a chronic NP state. Thus, preventing proteolysis of MBP may prove as a viable therapeutic strategy against neuropathic pain. It is tempting now to hypothesize that our findings broadly relate to pain associated with autoimmune demyelinating neuropathies and neurodegenerative disorders where the formation of cryptic MBP epitopes has also been documented [24,82].

## Competing interests

The authors declare no competing interests.

## Authors’ contributions

HL, YK, and IS performed animal surgeries and microinjections, behavioral testing, neuropathology, zymography, immunofluorescence and qPCR of the neuronal tissues, statistical analysis, and drafted the manuscript. SAS, AGR, and VSG carried out the MMP-9 purification, the biochemical characterization of MMP-9 proteolysis of MBP, mass spectrometry and proteomics, and drafted the manuscript. AVC and SB performed the microarray gene profiling experiments, the follow-on bioinformatics analyses, and drafted the manuscript. VIS and AYS conceived the study, participated in its design, coordinated the execution of the studies, and wrote the manuscript. All authors read and approved the final manuscript.

## Supplementary Material

Additional file 1 ** Table S1.**Unique proteins in CCI nerve listing, based on the 2D-LC-MS/MS of the sciatic nerve proteome after CCI (day 7). Click here for file

Additional file 2 ** Table S2.**Unique proteins in normal nerve listing based on the 2D-LC-MS/MS of the sciatic nerve proteome after sham operation (day 7). Click here for file

Additional file 3 **** Figure S1.**Autoimmune demyelination signaling in nerve after intraneural MBP84-104 injection.** Ingenuity Pathway Analysis of the gene expression used for generation the autoimmune demyelination signaling cascades at week 1 after intraneural MBP84-104 injection in wild-type rats and nude rats. Up-regulated expression of chemokine receptors and ligands are indicated in red. The intensity of red color corresponds to fold-change of expression level of respective genes. Activated T cells, producing CCR5 and CXCR3 (vertical rectangles) and monocytes, producing CCR5 and CCR1 receptors, are recruited into the intact nerve after MBP84-104 injection into the wild-type rats but not nude rats. CXCL9, CXCL10, and CXCL11 are ligands for CXCR3. CCL5, CCL3, and CCL4 are ligands for CCR5 and CCR1. Click here for file
