# Supplementary material for: Immunodominant fragments of myelin basic protein initiate T cell-dependent pain
Source: J Neuroinflammation. 2012 Jun 7;9:119. doi: 10.1186/1742-2094-9-119 (PMC3416717; doi:10.1186/1742-2094-9-119)

**Supplemental Figure 1. Autoimmune demyelination signaling in nerve after intraneural MBP84-104 injection.** Ingenuity Pathway Analysis of the gene expression used for generation the autoimmune demyelination signaling cascades at week 1 after intraneural MBP84-104 injection in wild-type rats and nude rats. Upregulated expression of chemokine receptors and ligands are indicated in red. The intensity of red color corresponds to fold-change of expression level of respective genes. Activated T cells, producing CCR5 and CXCR3 (vertical rectangles) and monocytes, producing CCR5 and CCR1 receptors, are recruited into the intact nerve after MBP84-104 injection into the wild-type rats but not nude rats. CXCL9, CXCL10 and CXCL11 are ligands for CXCR3. CCL5, CCL3 and CCL4 are ligands for CCR5 and CCR1.


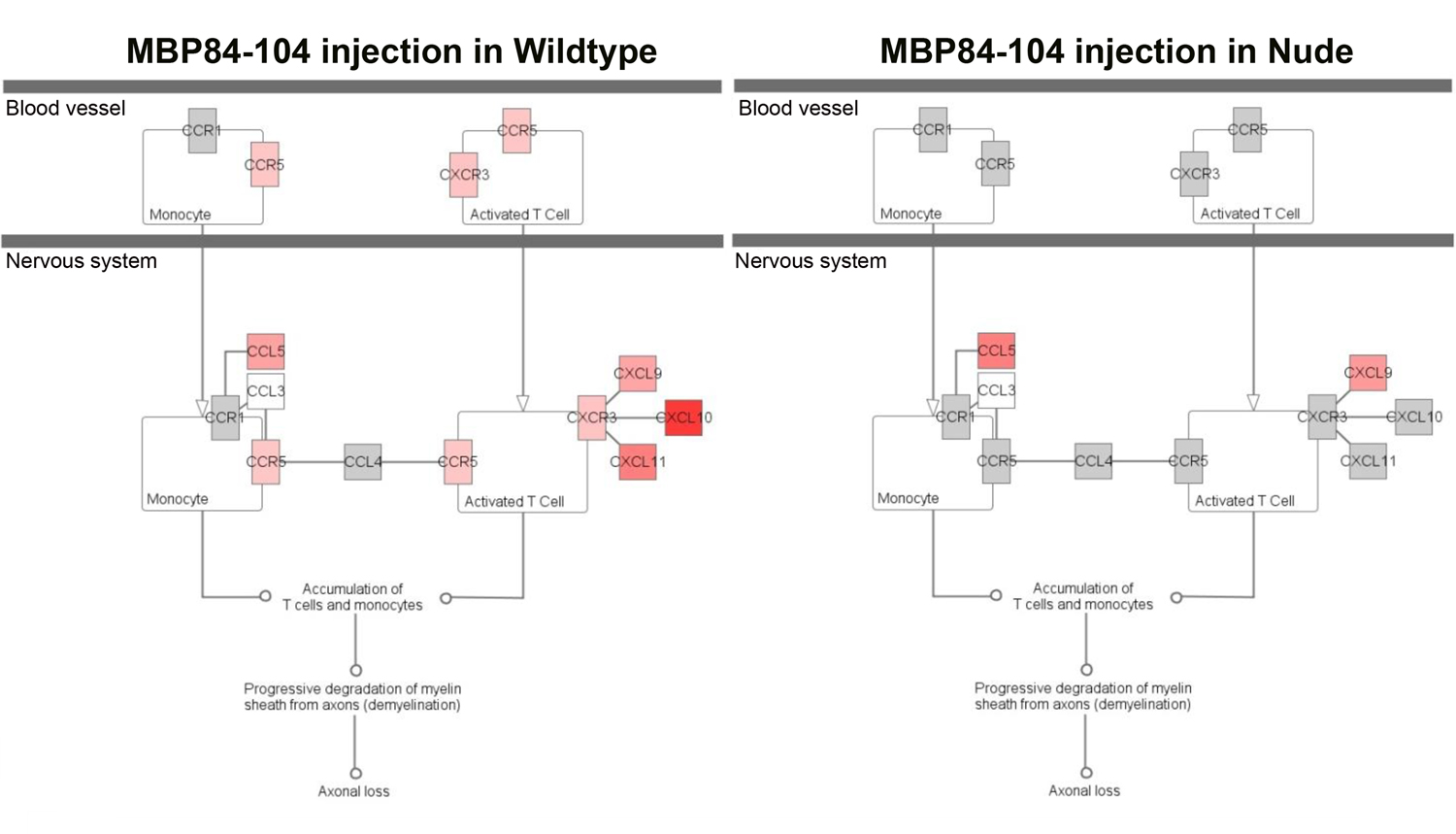

Supplement: Additional file 3 — Figure S1.Autoimmune demyelination signaling in nerve after intraneural MBP84-104 injection. Ingenuity Pathway Analysis of the gene expression used for generation the autoimmune demyelination signaling cascades at week 1 after intraneural MBP84-104 injection in wild-type rats and nude rats. Up-regulated expression of chemokine receptors and ligands are indicated in red. The intensity of red color corresponds to fold-change of expression level of respective genes. Activated T cells, producing CCR5 and CXCR3 (vertical rectangles) and monocytes, producing CCR5 and CCR1 receptors, are recruited into the intact nerve after MBP84-104 injection into the wild-type rats but not nude rats. CXCL9, CXCL10, and CXCL11 are ligands for CXCR3. CCL5, CCL3, and CCL4 are ligands for CCR5 and CCR1. [file 1742-2094-9-119-S3.doc]
